# Psychological and physiological correlates of childhood obesity in Taiwan

**DOI:** 10.1038/srep17439

**Published:** 2015-11-27

**Authors:** Kuo-Hsuan Chung, Hung-Yi Chiou, Yi-Hua Chen

**Affiliations:** 1 Department of Psychiatry, Taipei Medical University Hospital, 252 Wu-Hsing St., Taipei 110, Taiwan; 2 Psychiatric Research Center, Taipei Medical University Hospital, 252 Wu-Hsing St., Taipei 110, Taiwan; 3 Department of Psychiatry, School of Medicine, College of Medicine, Taipei Medical University, 250 Wu-Hsing St., Taipei 110, Taiwan; 4 School of Public Health, College of Public Health and Nutrition, Taipei Medical University, 250 Wu-Hsing St., Taipei 110, Taiwan

## Abstract

Evidence of associations between psychopathology and obesity in childhood remains inconsistent, and most studies have been conducted in Western countries. This study investigated psychological and physiological correlates of obesity in a community sample of children in Taiwan. In total, 302 children (157 overweight/obese and 145 healthy-weight children) were selected from first- and fourth-grade schoolchildren in eight elementary schools in 2009. These children participated in a comprehensive health examination, including a physical examination, blood sample analysis, and questionnaire administration. We found that regarding physiological characteristics, compared with the healthy-weight children, the overweight/obese children had significantly higher values for body fat estimated using the bioelectrical impedance method (*p* < 0.001), systolic blood pressure (*p* < 0.001), and diastolic blood pressure (*p* = 0.001); lower values for high-density lipoprotein (*p* < 0.001); and worse values for glutamic-pyruvic transaminase (*p* < 0.001), triglycerides (*p* < 0.001), and fasting blood glucose (*p* = 0.049). In logistic models adjusted for parental and child traits and physiological characteristics, children’s overweight/obesity was significantly associated with lower self-concept (odds ratio [OR] = 0.96, 95% confidence interval [CI] = 0.93–0.99) and less disruptive behavior (OR = 0.96, 95% CI = 0.92–0.99). Less disruptive behavior and the lack of a higher prevalence of anxiety and depression in childhood obesity appear to be a unique pattern in Taiwan that warrants further investigation.

Childhood obesity, similar to adulthood obesity, is a major global public health concern because of its wide geographic extent and high prevalence^1^,^2^. Obesity underlies a number of the most common illnesses seen in medical practice currently. Overweight and obese children are at a high risk of physiological abnormalities, including cardiovascular disease, metabolic syndrome^3^, nonalcoholic fatty liver disease, polycystic ovarian syndrome, and breathing abnormalities during sleep^4^. In addition to physical diseases, studies have shown that obese children and young people are prone to develop psychosocial distress, including depression, anxiety, and social withdrawal^5^,^6^,^7^ and tend to have a poor quality of life^1^,^8^ and behavioral problems^9^,^10^,^11^. However, evidence of associations between psychopathology and obesity in childhood remains inconsistent, and psychological and physiological correlates of childhood obesity remain unclear^11^, with variations according to gender, age, and contextual factors (such as familial eating, activity and nutritional patterns, and parenting attitudes), as well as race and ethnicity^12^,^13^,^14^,^15^.

There is a need of further research on psychological problems in obese children, as these problems may be exacerbated over time. A study showed that childhood obesity can be a precursor of adult depression^16^. Because obese children are much more likely to become obese adolescents and adults unless they maintain healthier patterns of exercise and eating^17^, a deeper understanding and early effective intervention should be emphasized in younger children. However, most studies have focused on the psychosocial aspects of childhood obesity in older children or adolescents^3^,^18^.

Most research on psychological correlates of childhood obesity has been conducted in Western countries. Although a trend of a Westernized lifestyle has been noted in Asia during the past decades, impacts of cultural differences on psychological heterogeneity among obese young people should not be ignored. Consistent with studies in Western countries, recent studies in Asia have demonstrated that obese children exhibit body dissatisfaction and lower self-esteem; however, no relationship was observed between obesity and higher levels of depressive symptoms in children^19^. Furthermore, the risk of depressive symptoms has been associated with a subjective sense of negative body image and not with the degree of obesity^14^. A strong relationship between self-perception and behavior may not be observed in Taiwanese children because of the emphasis on the Confucian ideology of filial piety and obedience that pervades Taiwanese culture, in which children are expected to respect and obey their parents and elders instead of expressing their true selves^20^. This practice may affect the psychological impact of obesity on children. For example, under the pressure of authoritarian parenting, children may be discouraged to use assertive and externalizing behaviors as coping mechanisms^20^. Furthermore, little research has been conducted on childhood obesity regarding psychological and physiological correlates. In doing this further research, it would be useful to also include further consideration from both a physiological standpoint (e.g., blood pressure, plasma glucose and triglyceride levels, birthweight) and a psychosocial standpoint (e.g., parental smoking, gender)^21^,^22^.

Therefore, in this study, we investigated the differences in physiological and psychological dimensions among schoolchildren with healthy and overweight or obese weight statuses in metropolitan Taipei, Taiwan. We hypothesize that compared with healthy-weight children, Taiwanese obese children have poor physiological characteristics (e.g., higher values for systolic blood pressure (SBP), triglycerides (TGs), or fasting blood glucose) and greater psychosocial distress (e.g., lower self-concept and more anxiety or depression). Patterns of association among Taiwanese children may differ from those observed among Western children.

## Methods

### Participants

In early 2009, a baseline screening was conducted for approximately 3,500 first (aged 6–7 years) and fourth (aged 9–10 years) graders from eight urban elementary schools in metropolitan Taipei, Taiwan. These schools were selected on the basis of their willingness to participate in a health promotion program. Preliminary information on birth date, height, weight, and blood pressure were collected. According to the World Health Organization criteria^23^, children were classified as having a healthy, overweight, or obese weight status depending on their body mass index (BMI) by gender and age.

During the baseline screening in the participating schools, all overweight and obese students were invited to take part in a more comprehensive health examination (a physical examination, blood sample analysis, and questionnaire administration) at a hospital. Some healthy-weight students also volunteered for this examination, with a weight status ratio of approximately 1:1. Finally, a total of 302 children (156 overweight or obese children and 145 healthy-weight children) completed the health examination undertaken at Taipei Medical University Hospital.

### Measurement of physiological characteristics

At the hospital, the children underwent a physical examination including the measurement of height, weight, and blood pressure. Body height and weight were recorded without shoes to the nearest 0.5 cm and 0.1 kg using a combined wall-mounted stadiometer and metric balance scale (NAGATA K-100/P-100), respectively. BMI was calculated using the formula: body weight (kg) divided by height squared (m^2^). SBP and diastolic blood pressure (DBP) were measured in duplicate on the right arm by trained personnel using an automated sphygmomanometer (TERUMO ES-P2000) after the children had been seated quietly for 5 min prior to the assessments. Body fat was estimated using the bioelectrical impedance method (Omron Body Fat Analyzer HBF-306). Blood samples were also collected from these children after a 12-h overnight fast for measurements of lipid levels, lipoproteins, serum liver enzymes, and fasting blood glucose.

### Measurement of psychological characteristics

The Beck Youth Inventories, second edition (BYI-II)^24^, was used to assess the children’s reported thoughts, feelings, and behaviors related to emotional and social dysfunction. Each inventory includes 20 questions that measure a child’s experiences in the five psychological domains of self-concept (assessing cognitions of competence, potency, and positive self-worth), anxiety (assessing worries about school performance, the future, negative reactions of others, and fears), depression (assessing negative thoughts about the self, life, and the future and feelings of sadness and guilt), anger (assessing thoughts of being treated unfairly by others and feelings of anger and hatred), and disruptive behavior (assessing thoughts and behaviors associated with conduct disorder and oppositional defiant behavior). Responses were scored using a 4-point Likert scale, with 0 indicating “never” and 3 indicating “always.” Total scores were summed for each inventory. The higher the scores were, the stronger the presentation of a particular psychological domain. In addition, the raw scores were converted (standardized) into T scores (with a mean of 50 and standard deviation of 10) to enable comparing a child’s score with that of other children of the same age.

While high internal consistency was indicated as Cronbach’s α coefficients ranged from 0.86 to 0.96 for all age groups on all scales, good test-retest reliability was reported to range from 0.74 to 0.93^25^. Validity was supported by correlations with other instruments assessing similar characteristics^25^. Furthermore, both the reliability and validity were established for the Chinese version of the BYI-II^26^. All Cronbach’s α coefficients were clearly >0.9 for the five inventories. Specifically for first to fourth graders in their norm group, the Cronbach’s α coefficients ranged from 0.92 to 0.94 for the five inventories. Test-retest reliability ranged from 0.64 to 0.81. Criterion-related validity was also supported for all five inventories.

Finally, parents filled out a questionnaire investigating risk factors for overweight and obesity in children. Questions included the parental BMI status, educational level, smoking status, and children’s birth history (i.e., maternal age when carrying the child, a low birthweight (birth weight < 2500 g), preterm birth, and cesarean delivery).

This study is a part of an integrated project on child obesity, which was approved by the Ethics Committee of Taipei Medical University Hospital. The methods were conducted in accordance with the approved guidelines. Each child’s parents provided written consent before the children were included in the study. The children’s consent for participation was also obtained.

### Data analysis

A Pearson’s χ^2^ test of significance was used to identify differences in the child, maternal, and paternal traits on the basis of the children’s weight status (healthy weight vs. overweight or obese). Psychological and physiological characteristics were separately examined on the basis of the children’s weight status. Specifically, regarding physiological characteristics, age, body fat, SBP, DBP, total cholesterol, high-density lipoprotein (HDL), low-density lipoprotein (LDL), and urea were normally distributed and were analyzed using independent-sample *t* tests. Other parameters, namely aspartate transaminase (AST), alanine aminotransferase (ALT), TGs, and fasting blood glucose, were not normally distributed and were analyzed using a Mann–Whitney *U*-test. Finally, multivariable logistic regression analyses were performed to examine the associations between children’s weight status (response variable) and the parental and child characteristics. The selection of potential confounding variables entered into the regression models was based on the combined data of preliminary analysis and results of previous studies (e.g., parental BMI^27^, maternal smoking^28^, and preterm birth^29^). In model fitting, the child’s sociodemographics and parental traits were considered first, and the child’s physiological and psychological characteristics were added separately. All parental and child characteristics were then considered in the final model. The backward stepwise model selection procedure, appropriate for selecting an interpretable model with the most valuable factors^30^, was used for variable selection. Interaction terms between gender and grade and other physiological and psychological characteristics that did not reach statistical significance were removed from the final analyses. SPSS version 15.0 (SPSS, Chicago, IL, USA) was used for the analysis. All tests of significance were two tailed, with the level of significance set to *p* < 0.05.

## Results

Table 1 shows a comparison of the healthy-weight students with the overweight or obese students in relation to child, maternal, and paternal traits. Ethnicity was not specifically assessed in our study, because the Taiwanese population is homogeneous and mainly dominated by the Han race (more than 90%). We found that compared with the healthy-weight children, the overweight or obese children were more likely to be male (*p* = 0.04). However, no significant differences were observed in the birth history, including maternal age when carrying the child, a low birthweight, preterm birth, and cesarean delivery, among children with different weight statuses (all *p* > 0.05). The overweight or obese children were more likely to have mothers who were overweight or obese (*p* < 0.01) and smoked (*p < *0.01) as well as fathers who were overweight or obese (*p* < 0.01).

### Children’s psychological characteristics according to weight status

The overweight or obese children had significantly lower scores for self-concept and disruptive behaviors compared with the healthy-weight children (self-concept: 49.7 ± 7.3 vs. 51.5 ± 7.5; *p* = 0.03; disruptive behaviors: 45.9 ± 8.6 vs. 48.1 ± 8.4; *p* = 0.03) (Table 2). No significant difference was observed in the domains of anxiety, depression, and anger between the healthy-weight and overweight or obese children.

### Children’s physiological characteristics according to weight status

Compared with the healthy-weight children, the overweight or obese children had significantly higher values for body fat (*p* < 0.001), SBP (*p* < 0.001), and DBP (*p* = 0.001) and significantly lower values for HDL (*p* < 0.001) (Table 2). The overweight or obese children also had worse ALT (*p* < 0.001), TG (*p* < 0.001), and fasting blood glucose (*p* < 0.05) values.

### The relationship between child weight status and psychological/physiological characteristics

In logistic models including parental and child traits, children’s overweight/obese status was significantly associated with maternal overweight/obesity (odds ratio [OR] = 2.90) and paternal overweight/obesity (OR = 2.20) (Model I, Table 3). The relationship between children’s weight status and their physiological and psychological characteristics was investigated separately. After adjustment for parental and child traits, childhood overweight and obesity were associated with higher levels of body fat, SBP, and urea (Model II) and with lower self-concept and less disruptive behavior (Model III). When physiological and psychological characteristics were considered simultaneously, childhood overweight and obesity remained associated with lower self-concept (OR = 0.96) and less disruptive behavior (OR = 0.96) in logistic models adjusted for parental and child traits (Model IV).

## Discussion

According to a review of relevant literature, this is the first study to investigate multiple psychological aspects, including self-concept, anxiety, depression, anger, and disruptive behavior, and obesity-related physiological correlates in school-age children in Taiwan. The major finding was that compared with a healthy-weight status, an overweight or obese status was associated with lower self-concept and less disruptive behavior in children, even after adjustment for parental and child traits. We found no evidence showing that they had more abnormalities in the psychological domains of anxiety, depression, and anger.

The construct of self-concept includes the subdomains of self-competence and self-worth, and self-worth is likely correlated with self-esteem^31^. Similar to obese Western children, the obese Taiwanese children had lower self-concept, suggesting no racial differences in the association between low self-esteem and obesity. Previous studies have reported modest associations between obesity and global self-esteem in Chinese and other ethnic groups, and body dissatisfaction seems to be associated with lower self-competence^13^,^19^,^32^,^33^. The inclusion of body image, self-competence, self-satisfaction, and attitudes toward others in the scales of self-concept in our measurement^26^ might partially explain the effect of body dissatisfaction on self-concept. Although the direction of causality between body dissatisfaction and self-esteem remains obscure, obesity may increase the risk of body dissatisfaction, which in turn impairs self-esteem^19^,^34^. In addition, the stigma attached to being obese may damage the self-concept of obese children owing to prejudices of healthy-weight children^1^,^10^,^15^,^35^, and negative parental opinions^36^. The relationship between obesity and psychological problems was suggested to emerge after children begin regular schooling, when they are at a higher risk of being exposed to attitudes of children and adults outside their families^6^.

In our community sample in Taiwan, we found that childhood obesity was associated with less disruptive behavior. This seems to be a unique phenomenon that should be further investigated. Studies in Western countries have described an association between obesity and increased likelihood of externalizing behavior in children^9^,^11^; however, the magnitude of this association and the starting age of this connection still remain unclear^9^. In addition, evidence suggested that the impulsivity associated with disruptive behavior in childhood obesity could be related to dopamine dysfunction, poor inhibitory control, and reward sensitivity^11^. However, previous studies have indicated that, in Chinese culture, children are more likely to have an increased capacity to use somatization or internalization as a means of coping^37^. Confucian ideology strongly pervades Taiwanese culture, and its focus on obedience and respect strongly discourages the use of assertive and externalizing behaviors as coping mechanisms^20^. With the increasing trend of bullying in Taiwan^38^, obese children may encounter more discrimination owing to social stigmas; therefore, obese ethnic Chinese (i.e., Taiwanese) children may be prone to use somatization rather than present disruptive behaviors. By contrast, a recent study suggested that, in Taiwan, authoritarian parenting styles may affect parental child-feeding practices^39^. To distract children from their emotional or behavioral problems, Taiwanese parents may use food as a reward or may allow them to engage in sedentary pastimes such as watching television and playing videogames^40^. Such behavioral management strategies might result in childhood obesity. Therefore, cultural differences may account for the less disruptive behavior in our sample. To verify the association between childhood obesity and disruptive behavior, longitudinal follow-up or additional similar studies should be performed in Chinese and Taiwanese communities.

Previous studies reported an association of obesity with anxiety and depression^5^,^6^,^7^. In our study, although the sample size may be a reason for finding no association between childhood obesity and depression or anxiety, the finding may partially be explained by cultural factors. Western research has revealed that “weight stigma” can emerge in a subtle or overt form, leading to prejudice, discrimination, victimization, and devalued social identity, which might mediate the relationship between depression and obesity^15^. Although stigmatization of childhood obesity appears to be a worldwide public health issue, Chinese culture has traditionally regarded plumpness, particularly in children, as relatively acceptable, healthy, or attractive^19^,^41^. In accordance with our findings, studies from Korea have indicated no relationship between obesity and high levels of depressive symptoms in children^14^,^19^, suggesting that Asian culture might at least protect children from depression. Regarding biological factors, it was proposed that depression and body fat regulation share genetic factors and have common monoamines and peptides (including serotonin, norepinephrine, dopamine, neuropeptide Y, and corticotropin-releasing hormone)^11^. However, the exact mechanisms underlying the associations between childhood obesity and mood dysregulation remain to be clarified.

As expected and consistent with previous studies^3^, the overweight or obese children had more physiological abnormalities than did the healthy-weight children, such as abnormal lipids, impaired liver function, and higher blood pressure and fasting blood glucose. Obesity is regarded as a chronic, low-grade systemic inflammatory state^1^. Inflammatory or immune markers might be used in predicting the severity of obesity-related illness^42^ if future studies of the pathogenesis or pathophysiology of obesity-related inflammation confirm the association between these biomarkers and childhood obesity.

Our study had several limitations. First, the study had a cross-sectional design. Longitudinal follow-up studies are required to clarify causal relationships between childhood obesity and psychological distress. Because our sample was a small subgroup of a much larger population, it is unclear whether the findings can be applied to the general population. Specifically, calculating the sample size in our study would yield statistical powers of 0.65 to 0.95, depending on various physiological and psychological characteristics used among children with healthy and overweight or obese weight statuses. Second, in our study, schools were selected on the basis of their willingness to participate in a health promotion program, and they are located in Taipei City, which is one of the five major cities with high health care accessibility in Taiwan. These schools might consider students’ health issues a higher priority, possibly diminishing the differences between the healthy-weight and overweight or obese children. Ethnicity was not specifically assessed in our study, because the Taiwanese population is homogeneous and mainly dominated by the Han race. In addition, because the students were sampled in an urban area, our sample is a limited representation of the entire Taiwanese population.

Third, the rating scale used in this study was self-reported. Measurements of psychological characteristics may be incomplete because of problems with the completeness of reporting and may be subject to socially desirable effects and the participant’s interpretation. In addition, our findings on self-reported psychological characteristics, particularly by first graders, should be interpreted with caution, although researchers have suggested that first graders’ self-report of emotional disturbances, such as anxiety, might have clinical significance on the basis of their findings on the stability, caseness, prevalence, and prognostic value of self-reported measures^43^,^44^; moreover, the reliability and validity were established for the Chinese version of the BYI-II^26^. A multi-informant approach may be considered in the future to provide a more accurate description of children’s problems in various contexts, with the simultaneous consideration of difficulties and discordance inherent in various reporters^45^. Finally, research on childhood obesity and comparison of data among countries may be problematic because of the lack of an internationally accepted measure of obesity^3^. Therefore, the results should be generalized to other populations with caution.

## Conclusions

The key physiological finding of our study is that urban Taiwanese children in the first and fourth grades show signs of weight-related comorbidity even at this young age. Regarding psychological correlates, our findings indicate that childhood obesity is associated with lower self-concept. Less disruptive behavior and the lack of a higher prevalence of anxiety and depression in childhood obesity appear to be a unique pattern, which deserves further consideration. Although emotional and disruptive behaviors might not be a major issue in young overweight and obese children, careful attention should be paid to low self-esteem, consistently observed in our and Western studies, because of its adverse effects on mental health^1^,^5^,^6^,^7^,^8^,^9^,^10^,^11^. With increasing trends of obese children in both Western and Eastern countries, it is critical to recognize unfavorable values of certain physiological and psychological characteristics among overweight and obese students as young as 6–7 years and 9–10 years (first and fourth graders, respectively) to promote the physical and mental well-being of children in the current obesogenic environment.

## Additional Information

**How to cite this article**: Chung, K.-H. *et al*. Psychological and physiological correlates of childhood obesity in Taiwan. *Sci. Rep*. **5**, 17439; doi: 10.1038/srep17439 (2015).

## Figures and Tables

**Table 1:** Comparison of healthy-weight and overweight or obese children in relation to maternal, paternal, and child traits (*n* = 302).

	Healthy weight (*n* = 145)	Overweight/obesity (*n* = 157)	*p*value
**Child traits**			
Gender			0.004^a^
Male	68 (46.9%)	92 (58.6%)	
Female	77 (53.1%)	65 (41.4%)	
Grade			0.66^a^
First	72 (49.7%)	74 (47.1%)	
Fourth	73 (50.3%)	83 (52.9%)	
Age (years)	8.8 (1.7)	8.9 (1.4)	0.63^b^
**Birth history**			
Maternal age when carrying the child			0.60^a^
<35 years	117 (81.8%)	127 (84.1%)	
≥35 years	26 (18.2%)	24 (15.9%)	
Low birthweight			0.82^a^
Yes	11 (7.9%)	13 (8.6%)	
No	129 (92.1%)	138 (91.4%)	
Birthweight (g)	3158 (500)	3213 (509)	0.35^b^
Preterm birth			0.21^a^
Yes	9 (7.4%)	16 (12.2%)	
No	112 (92.6%)	115 (87.8%)	
Cesarean delivery			0.19^a^
Yes	52 (36.1%)	67 (43.5%)	
No	92 (63.9%)	87 (56.5%)	
**Maternal traits**			
BMI status			< 0.001^a^
Healthy weight	111 (78.7%)	85 (56.7%)	
Overweight/obese	30 (21.3%)	65 (43.3%)	
Educational level			0.13^a^
Lower than high school	6 (4.2%)	10 (6.6%)	
High school	61 (43.0%)	79 (52.0%)	
College or higher	75 (52.8%)	63 (41.4%)	
Smoking status			0.002^a^
No	138 (97.9%)	133 (86.9%)	
Quit smoking	1 (0.7%)	3 (2.0%)	
Current smoker	2 (1.4%)	17 (11.1%)	
**Paternal traits**			
BMI status			0.002^a^
Healthy weight	60 (44.8%)	40 (27.2%)	
Overweight/obese	74 (55.2%)	107 (72.8%)	
Educational level			0.06^a^
Lower than high school	6 (4.3%)	17 (11.0%)	
High school	49 (35.3%)	59 (38.3%)	
College or higher	84 (60.4%)	78 (50.7%)	
Smoking status			0.66^a^
No	70 (50.7%)	71 (45.5%)	
Quit smoking	12 (8.7%)	16 (10.3%)	
Current smoker	56 (40.6%)	69 (44.2%)	

Data are n (%), except age and birthweight data that are mean (SD). Note that total counts may vary because

of missing data.

^a^Results were obtained using a χ^2^ test.

^b^Results were obtained using a *t* test.

**Table 2:** Psychological and physiological characteristics of children according to weight status (*n* = 302).

	Healthy weight (*n* = 145)	Overweight/obese (*n* = 157)	*p* value
Age (years)	8.8 (1.7)	8.9 (1.4)	0.63^a^
Psychological characteristics			
Self-concept	51.5 (7.5)	49.7 (7.3)	0.03^a^
Anxiety	46.9 (8.4)	47.4 (8.4)	0.58^a^
Depression	45.5 (8.9)	45.8 (9.3)	0.75^a^
Anger	47.8 (8.8)	46.4 (9.1)	0.16^a^
Disruptive behavior	48.1 (8.4)	45.9 (8.7)	0.03^a^
Physiological characteristics			
Body fat	18.3 (5.5)	33.4 (6.5)	< 0.001^a^
SBP (mmHg)	102.1 (9.7)	108.3 (12.9)	< 0.001^a^
DBP (mmHg)	60.2 (7.6)	63.7 (10.4)	0.001^a^
T-Chol (mg/dL)	174.4 (29.2)	173.3 (31.4)	0.74^a^
HDL (mg/dL)	62.6 (11.3)	56.7 (12.2)	< 0.001^a^
LDL (mg/dL)	98.1 (22.8)	102.0 (27.6)	0.18^a^
Urea (mg/dL)	12.0 (3.0)	12.4 (2.6)	0.25^a^
AST (IU/L)	15–45 (25)	14–124 (23)	0.12^b^
ALT (IU/L)	5–95 (13)	5–271 (16)	< 0.001^b^
TGs (mg/dL)	25–204 (54)	26–356 (70)	< 0.001^b^
Fasting blood glucose (mg/dL)	71–173 (86)	69–363 (88)	0.05^b^

Data are mean (SD) or range (median).

SBP, systolic blood pressure; DBP, diastolic blood pressure; T-Chol, total cholesterol; HDL, high-density lipoprotein; LDL, low-density lipoprotein; AST, aspartate transaminase; ALT, alanine aminotransferase; TGs, triglycerides.

^a^Results were obtained using a *t* test.

^b^Results were obtained using a Mann–Whitney U-test.

**Table 3:** The relationship between child weight status and psychological/physiological characteristics.

Variable	Model I^a^	Model II^b^	Model III^c^	Model IV^d^
Parental and child traits				
Gender				
Female	1.00	1.00	1.00	1.00
Male	2.35^**^ (1.29–4.31)	1.10 (0.42–2.83)	1.44 (0.90–2.30)	1.61 (0.89–2.92)
Grade				
First	1.00	1.00	1.00	1.00
Fourth	0.88 (0.49–1.58)	0.59 (0.22–1.56)	1.19 (0.75–1.91)	1.14 (0.63–2.06)
Preterm birth				
No	1.00			
Yes	2.34 (0.86–6.35)			
Maternal smoking				
No	1.00			
Yes	16.18^**^ (2.01–130.41)			
Maternal weight				
Healthy	1.00			1.0
Overweight/obese	2.90^**^ (1.54–5.45)			3.14^***^ (1.70–5.79)
Paternal weight				
Healthy	1.00			1.0
Overweight/obese	2.20^*^ (1.16–4.17)			2.44^**^ (1.31–4.55)
Child physiological characteristics				
Body fat	—	1.60^***^ (1.42–1.81)	—	
Systolic blood pressure	—	1.04^*^ (1.00–1.09)	—	
Urea	—	1.21^*^ (1.00–1.45)	—	1.01 (1.00–1.02)
Low-density lipoprotein	—	0.98 (0.96–1.00)	—	1.02 (0.93–1.13)
Child psychological characteristics				
Self-concept	—	—	0.96^**^ (0.93–0.99)	0.96^*^ (0.92–0.99)
Disruptive behavior	—	—	0.96^**^ (0.94–0.99)	0.96^*^ (0.93–0.99)

Data are odds ratio of being overweight or obese and associated 95% confidence intervals.

*p < 0.05; **p < 0.01; ***p < 0.001.

^a^Model I includes parental and child characteristics, and the backward stepwise model selection procedure was used for variable selection.

^b^Model II includes parental and child characteristics and child physiological characteristics, and the backward stepwise model selection procedure was used for variable selection.

^c^Model III includes parental and child characteristics and child psychological characteristics, and the backward stepwise model selection procedure was used for variable selection.

^d^Model IV includes parental and child characteristics and child physiological and psychological characteristics, and the backward stepwise model selection procedure was used for variable selection.
